# Global Profile of Drug Resistance Related to *Helicobacter pylori* Infection in Children: A Systematic Review and Meta‐Analysis

**DOI:** 10.1002/hsr2.71238

**Published:** 2025-09-14

**Authors:** Shaho Menbari, Sara Kamal Shahsavar, Masoud Keikha, Mohsen Karbalaei

**Affiliations:** ^1^ Department of Pathology and Medical Laboratory Sciences, Faculty of Para Medicine Kurdistan University of Medical Sciences Sanandaj Iran; ^2^ Antimicrobial Resistance Research Center, Bu‐Ali Research Institute Mashhad University of Medical Sciences Mashhad Iran; ^3^ Department of Microbiology and Virology, School of Medicine Mashhad University of Medical Sciences Mashhad Iran; ^4^ Tropical and Communicable Diseases Research Center Iranshahr University of Medical Sciences Iranshahr Iran; ^5^ Department of Microbiology and Virology, School of Medicine Iranshahr University of Medical Sciences Iranshahr Iran; ^6^ Department of Microbiology and Virology, School of Medicine Jiroft University of Medical Sciences Jiroft Iran; ^7^ Bio Environmental Health Hazards Research Center Jiroft University of Medical Sciences Jiroft Iran

**Keywords:** antimicrobial resistance, children, *Helicobacter pylori*, treatment

## Abstract

**Background and Aims:**

The increasing prevalence of antibiotic‐resistant *Helicobacter pylori* (*H. pylori*) strains represents a critical ‎impediment to successful eradication therapy in both pediatric and adult populations. This ‎meta‐analysis aimed to determine the current global landscape of primary antibiotic resistance ‎in bacterial isolates obtained from children.

**Methods:**

A systematic literature search was conducted across ISI Web of Science, PubMed, Scopus, ‎and Google Scholar, encompassing the period from the inception of each database up to ‎December 2021. Eligible studies reporting primary antibiotic resistance in *H. pylori* isolates ‎from children worldwide were included. Resistance rates were expressed as percentages with ‎corresponding 95% confidence intervals. Statistical analysis was performed using ‎Comprehensive Meta‐Analysis 2.2.

**Results:**

One hundred eleven teens were included in this meta‐analysis, and 36,021 isolates of this bacterium were evaluated. The resistance rate was reported 25.6%, 30.9%, 2.5%, 2.0%, 12.1%, 6.9%, 1.9%, 0.5%, and 9.1%, for clarithromycin, metronidazole, amoxicillin, tetracycline, levofloxacin, ciprofloxacin, furazolidone, nitrofurantoin, and rifampin respectively. Furthermore, the pooled prevalence of primary multidrug resistant isolates was 4.5%.

**Conclusion:**

This meta‐analysis reveals a significant global burden of primary resistance to clarithromycin ‎and metronidazole in pediatric *H. pylori* isolates, with evidence of increasing resistance over ‎time. Conversely, resistance rates to amoxicillin, tetracycline, levofloxacin, ciprofloxacin, ‎furazolidone, nitrofurantoin, and rifampin remained low. Consequently, therapeutic regimens ‎incorporating clarithromycin and metronidazole should be carefully considered and ‎potentially avoided in regions exhibiting resistance rates exceeding 20%.

**Abbreviations**
ADM: agar dilution method
AST: antimicrobial susceptibility testing
CI: confidence intervals
CLSI: clinical and Laboratory Standards Institute
DDT: disk diffusion test
ESPGHAN: European Society for Pediatric Gastroenterology Hepatology and Nutrition
FISH: fluorescence in situ hybridization
*H. pylori*
: *Helicobacter pylori*
MDR: multidrug resistant
NASPGHAN: North American Society for Pediatric Gastroenterology, Hepatology and Nutrition
PCR: polymerase chain reaction
PPIs: proton pump inhibitors
PRISMA: preferred reporting items for systematic reviews and meta‐analyzes

## 1 Background

*Helicobacter pylori* (*H. pylori*) is a Gram‐negative, spiral bacterium characterized by urease ‎activity and lophotrichous flagella, enabling motility under microaerophilic conditions within ‎the human gastric environment [1]. This pathogen establishes persistent colonization in the ‎gastric mucosa of over two billion individuals globally, with acquisition typically occurring in ‎childhood. In developing nations, such chronic infections frequently progress to severe ‎gastroduodenal sequelae, including peptic ulcer disease and gastric adenocarcinoma [2, 3]. ‎Notably, contemporary epidemiological investigations of *H. pylori* seroprevalence indicate a ‎declining trend in infection rates across both developing and developed countries [4]. For ‎instance, a study in Japan (1991–2017) demonstrated a decrease in *H. pylori* infection rates in ‎children from approximately 10% for those born in 1985% to 3% for those born in early 2011 ‎‎ [5]. Similar significant reductions in *H. pylori* prevalence have been observed over time in ‎European populations, mirroring the trends in Asia [6, 7]. ‎

Compared to adults, severe gastroduodenal manifestations are less common in children, and ‎emerging evidence even suggests a potential immunological benefit associated with *H. pylori* ‎infection during childhood [8]. While *H. pylori* infection in children induces microscopic ‎gastric inflammation, the majority of infected children remain asymptomatic and do not ‎typically experience functional gastrointestinal disorders such as recurrent abdominal ‎complications [9]. Consequently, the decision to pursue *H. pylori* eradication in children ‎necessitates a careful assessment of the individual benefit for each child [10]. Regrettably, the ‎escalating issue of *H. pylori* antibiotic resistance has diminished eradication efficacy and is ‎now a primary determinant of treatment failure [11]. Given the critical role of clarithromycin ‎in treatment outcomes, the World Health Organization (WHO) has recently issued warnings ‎regarding increasing *H. pylori* resistance to this antibiotic [12, 13]. Furthermore, therapeutic ‎options for pediatric patients are more restricted, and the absence of an effective vaccine ‎against *H. pylori* infection exacerbates this challenge [4, 14]. ‎

Current guidelines from the European Society for Pediatric Gastroenterology Hepatology and ‎Nutrition (ESPGHAN) and the North American Society for Pediatric Gastroenterology, ‎Hepatology and Nutrition (NASPGHAN) recommend several considerations for managing *H. ‎pylori* infection in children: (1) antibiotic selection should be guided by susceptibility testing; ‎‎(2) a 14‐day treatment duration with strict adherence is advised; (3) clarithromycin use should ‎be limited to susceptible strains; and (4) treatment success should be confirmed 4 to 8 weeks ‎post‐therapy [10]. The updated ESPGHAN/ASPGHAN guidelines designate a 14‐day ‎bismuth‐based regimen as the first‐line treatment in the absence of antimicrobial susceptibility ‎testing (AST) results; however, standard triple therapy is the preferred initial approach if ‎bismuth is unavailable [10, 15]. ‎ In some parts of the world, gastroenterologists also treat *H. pylori* infection according to the European and American guidelines, which mighty lead to treatment failure in some areas due to differences in the characteristics of *H. pylori* strains in different parts of the world; in South Korea, endoscopy is recommended to determine AST in cases of treatment failure [16]. According to AST, two antibiotics plus maximum tolerable dosage of proton pump inhibitors (PPIs) and bismuth salt are administrated for 14 days [17].

Adjunctive probiotic administration alongside antibiotics has demonstrated potential for ‎improving *H. pylori* eradication rates [18, 19]. A meta‐analysis by Fang et al. indicated that ‎probiotic supplementation can reduce *H. pylori*‐associated diarrhea by up to 13% [20]. ‎However, the increasing prevalence of antibiotic resistance in recent years poses a significant ‎obstacle to successful *H. pylori* eradication. This burden is particularly concerning in Asian ‎countries, where the eradication rate of standard clarithromycin‐based triple therapy falls ‎below 80%. Therefore, continuous monitoring of *H. pylori* antibiotic resistance prevalence ‎and temporal trends is crucial for establishing optimal therapeutic strategies in children [11]. ‎While numerous studies have investigated *H. pylori* antibiotic resistance, many have focused ‎primarily on adolescent populations. To the authors' knowledge, a comprehensive systematic ‎review examining the trend of *H. pylori* antibiotic resistance specifically in children remains ‎lacking. The present study aimed to evaluate the prevalence of primary *H. pylori* antibiotic ‎resistance in children and to assess its temporal trends over the past three decades.

## 2 Methods

### 2.1 Search Strategy and Evaluation Criteria

This systematic review and meta‐analysis were conducted in accordance with the Preferred Reporting Items for Systematic Reviews and Meta‐Analyzes (PRISMA) 2020 guidelines. Because this study is a systematic review and meta‐analysis of previously published studies, it does not involve the collection or use of individual patient data. Accordingly, institutional review board approval and informed consent were not required. The aim was to investigate the global profile of antibiotic resistance associated with *Helicobacter pylori* infection in children. A comprehensive literature search was conducted to identify relevant studies published up to December 2021 in the following databases: PubMed, Scopus, ISI Web of Science, and Google Scholar, without any restrictions on language or publication year. The search strategy combined Medical Subject Headings (MeSH) and free‐text terms using appropriate Boolean operators.

The literature search strategy was customized for each database using a combination of MeSH terms and free‐text keywords related to *H. pylori*, antibiotic resistance, and pediatric populations; the specific search syntaxes for PubMed, Scopus, Web of Science, and Google Scholar are detailed in Table 1.

**Table 1:** Search strategies based on the syntax and indexing systems of each database.

Database	Search syntax	Retrieved articles
PubMed	(“*Helicobacter pylori*”[MeSH] OR “*H. pylori*”[tiab] OR “*Helicobacter pylori*”[tiab]) AND (“Drug Resistance, Bacterial”[MeSH] OR “antibiotic resistance”[tiab] OR “antimicrobial resistance”[tiab]) AND (“Child”[MeSH] OR “Pediatrics”[MeSH] OR “children”[tiab] OR “pediatric”[tiab])	573
Scopus	TITLE‐ABS‐KEY(“*Helicobacter pylori*” OR “*H. pylori*”) AND TITLE‐ABS‐KEY(“antibiotic resistance” OR “antimicrobial resistance”) AND TITLE‐ABS‐KEY(“children” OR “pediatric” OR “child”)	281
Web of science	TS = (“*Helicobacter pylori*” OR “*H. pylori*”) AND TS = (“antibiotic resistance” OR “antimicrobial resistance”) AND TS = (“children” OR “child” OR “pediatric”)	128
Google scholar	“*Helicobacter pylori*” AND “antibiotic resistance” AND (children OR pediatric OR child)	804

To ensure completeness, reference lists of included studies were also manually screened. Although no language restrictions were applied during the search process, the inclusion of non‐English articles was managed as follows: studies published in languages other than English were initially screened by title and abstract using automated translation tools (e.g., Google Translate). If potentially eligible, the full texts were translated either through professional translation services or with the assistance of bilingual researchers familiar with medical terminology. This ensured that all relevant studies, regardless of language, were considered for inclusion. Inclusion criteria included: (1) Original articles reporting primary data on *H. pylori* antibiotic resistance patterns; (2) Studies conducted in pediatric populations; (3) Isolation of *H. pylori* from human clinical samples; (4) Use of CLSI‐based methods for antimicrobial susceptibility testing; (5) Cross‐sectional descriptive design. Also, repeated articles, in vitro or in vivo studies, Reviews, case reports, letters, editorials, conference abstracts, Studies involving nonhuman samples or duplicated patient populations, Articles with unclear methodology or insufficient data for extraction were considered as exclusion criteria. To evaluate publication bias, we applied both visual (i.e., Funnel plot) and statistical methods (i.e., Egger's regression and Begg's test). Two independent reviewers screened all titles, abstracts, and full texts. Discrepancies were resolved through discussion and consensus.

### 2.2 Quality Assessment and Data Extraction

The methodological quality of the included studies was evaluated using the Joanna Briggs Institute (JBI) checklist [21]. This checklist assesses various aspects, including population representativeness, research objectives clarity, sample collection methodology, appropriateness of statistical analysis, and the specific methods employed. Studies achieving a quality score of at least seven were included in the final analysis. To extract the necessary data, the full texts of eligible studies were meticulously reviewed. The extracted information comprised the first author, publication year, study location, number of participants, type of antibiogram method utilized, number of *H. pylori* isolates analyzed, the frequency of resistance to specific antibiotics (clarithromycin, metronidazole, amoxicillin, tetracycline, levofloxacin, ciprofloxacin, nitrofurantoin, furazolidone, rifampin), and the prevalence of multidrug‐resistant (MDR) *H. pylori* in children presenting with upper gastrointestinal symptoms. These data were systematically compiled in Table 2 [22, 23, 24, 25, 26, 27, 28, 29, 30, 31, 32, 33, 34, 35, 36, 37, 38, 39, 40, 41, 42, 43, 44, 45, 46, 47, 48, 49, 50, 51, 52, 53, 54, 55, 56, 57, 58, 59, 60, 61, 62, 63, 64, 65, 66, 67, 68, 69, 70, 71, 72, 73, 74, 75, 76, 77, 78, 79, 80, 81, 82, 83, 84, 85, 86, 87, 88, 89, 90, 91, 92, 93, 94, 95, 96, 97, 98, 99, 100, 101, 102, 103, 104, 105, 106, 107, 108, 109, 110, 111, 112, 113, 114, 115, 116, 117, 118, 119, 120, 121, 122, 123, 124, 125, 126, 127, 128, 129, 130, 131, 132, 133, 134, 135, 136, 137, 138]. Data extraction was performed independently by two authors, and any disagreements were adjudicated by a third author.

**Table 2:** Antimicrobial resistance profiles of *H. pylori* in children.

First author	Year	Area	Patients (*n*)	Method	HP Strains	CLA	MTZ	AMO	TET	LVX	CIP	NIT	FUR	RIF	MDR	Reference
Loo	1992	Canada	18	ADM	18	NA	0	0	NA	NA	0	NA	NA	NA	NA	[22]
Rozynek	1997	Poland	130	E‐test	130	16.9	51.5	0	0	NA	0.8	NA	NA	NA	NA	[23]
Mentis	1999	Greece	36	ADM	36	5.5	28.0	0	0	0	NA	NA	NA	NA	NA	[24]
Cabrita	2000	Portugal	58	E‐test	58	44.8	19.0	0	0	NA	0	NA	NA	NA	NA	[25]
Tolia	2000	USA	31	E‐test	22	50.0	45.4	4.4	0	NA	NA	NA	NA	NA	NA	[26]
Kalach	2001	France	150	E‐test	150	21.0	43.0	0	NA	NA	NA	NA	NA	NA	NA	[27]
Glupczynski	2001	Europe	1274	E‐test	1274	9.9	33.1	0.8	NA	NA	NA	NA	NA	NA	NA	[28]
Kalach	2001	France	61	E‐test	61	18.0	NA	NA	NA	NA	NA	NA	NA	NA	NA	[29]
Torres	2001	Mexico	51	E‐test	51	21.6	78.4	15.7	NA	NA	NA	NA	NA	NA	NA	[30]
Fangrat	2001	Poland	98	E‐test	98	23.5	NA	NA	NA	NA	NA	NA	NA	NA	NA	[31]
Yang	2001	Taipei	245	E‐test	67	18.0	9.0	NA	NA	NA	NA	NA	NA	NA	NA	[32]
Kalach	2001	France	150	E‐test	150	21.0	43.0	0	NA	NA	NA	NA	NA	NA	NA	[33]
López‐Brea	2001	Spain	246	ADM	246	21.13	23.01	0	NA	NA	NA	NA	NA	NA	NA	[34]
Schmidt	2002	Germany	149	FISH	75	21.9	NA	NA	NA	NA	NA	NA	NA	NA	NA	[35]
Taneike	2002	Japan	14	DDT	14	42.9	0	0	0	NA	NA	NA	NA	NA	NA	[36]
Boyanova	2002	Bulgaria	115	ADM	114	12.4	15.8	0	3.1	NA	6.0	NA	NA	NA	1.2	[37]
Rozynek	2002	Poland	259	E‐test	259	19.3	37	0	0.4	NA	NA	NA	NA	NA	NA	[38]
Kato	2002	Japan	48	E‐test	48	29.0	24.0	0	NA	NA	NA	NA	NA	NA	NA	[39]
Crone	2003	Austria	117	E‐test	98	20.4	16.0	0	NA	NA	NA	NA	NA	NA	NA	[40]
Alarcon	2003	Argentina	96	ADM	96	29.1	23.9	0	NA	NA	NA	NA	NA	NA	NA	[41]
Romaniszyn	2003	Poland	45	E‐test	45	16.0	18.0	0	0	NA	NA	NA	NA	NA	NA	[42]
Rerksuppapho	2003	Australia	23	E‐test	23	8.7	43.5	0	0	NA	NA	NA	NA	NA	NA	[43]
Boyanova	2004	Bulgaria	186	ADM	186	11.9	14.5	0	3.3	NA	NA	NA	NA	NA	NA	[44]
Fujimura	2004	Japan	55	E‐test	55	21.8	9.1	0	NA	5.5	NA	NA	NA	NA	NA	[45]
Sherif	2004	Egypt	48	E‐test	48	4.0	0	2.0	NA	NA	2.0	NA	NA	NA	NA	[46]
Maciorkowska	2004	Poland	50	E‐test	50	25.0	NA	NA	NA	NA	NA	NA	NA	NA	NA	[47]
Goscinlak	2004	Poland	409	E‐test	409	8.6	35.2	NA	NA	NA	NA	NA	NA	NA	NA	[48]
Chen	2004	China	108	E‐test	108	55.5	NA	NA	NA	NA	NA	NA	NA	NA	NA	[49]
Chen	2004	China	115	E‐test	44	18.2	31.8	9.1	NA	NA	NA	NA	NA	NA	NA	[50]
Falsafi	2004	Iran	70	ADM	70	75.0	79.0	58.0	NA	65.0	NA	NA	NA	NA	NA	[51]
Fangrat	2005	Poland	179	E‐test	179	28.0	40.0	0	0	NA	NA	NA	NA	NA	NA	[52]
Booka	2005	Japan	23	PCR	16	31.0	NA	NA	NA	NA	NA	NA	NA	NA	NA	[53]
Lopes	2005	Portugal	109	E‐test	109	39.4	16.5	0	0	NA	4.5	NA	NA	NA	NA	[54]
Mard	2005	France	60	E‐test	60	5.0	15.0	0	NA	NA	NA	NA	NA	NA	NA	[55]
Faber	2005	Israel	105	E‐test	105	15.2	31.4	NA	NA	NA	NA	NA	NA	NA	NA	[56]
Raymond	2005	France	14	E‐test	14	57.1	28.5	NA	NA	NA	NA	NA	NA	NA	NA	[57]
Elitsur	2006	USA	16	FISH	16	12.5	NA	NA	NA	NA	NA	NA	NA	NA	NA	[58]
Koletzko	2006	Europe	1233	E‐test	1233	20.0	23.0	0.6	NA	NA	NA	NA	NA	NA	NA	[59]
Boyanova	2006	Bulgaria	28	ADM	28	12.5	15.0	1.5	3.4	NA	NA	NA	NA	NA	NA	[60]
Arenz	2006	Germany	58	E‐test	58	9.0	16.0	NA	NA	NA	NA	NA	NA	NA	NA	[61]
Siavashi	2006	Iran	51	DDT	51	5.9	37.0	5.9	2.0	NA	NA	NA	0	NA	NA	[62]
Lottspeich	2007	Germany	100	PCR	46	63.0	NA	NA	NA	NA	NA	NA	NA	NA	NA	[63]
Rafeey	2007	Iran	100	E‐test	100	16.0	95.0	59.0	5.0	NA	7.0	NA	9.0	NA	NA	[64]
Fallahi	2007	Iran	24	DDT	24	4.16	54.16	8.33	0	NA	NA	NA	0	NA	NA	[65]
Kalach	2007	France	377	E‐test	377	22.8	36.7	0	NA	NA	NA	NA	NA	NA	NA	[66]
Hu	2007	China	127	E‐test	127	NA	44.8	NA	NA	NA	NA	NA	NA	NA	NA	[67]
Raymond	2007	French	217	E‐test	217	23.0	NA	NA	NA	NA	NA	NA	NA	NA	NA	[68]
Boyanova	2008	Bulgaria	75	E‐test	75	18.7	16.0	0	2.7	NA	6.8	0	NA	NA	0	[69]
Caristo	2008	Italy	68	FISH	68	37.0	NA	NA	NA	NA	NA	NA	NA	NA	NA	[70]
Tanuma	2009	Thailand	284	PCR	120	29.2	NA	NA	NA	NA	NA	NA	NA	NA	NA	[71]
Boyanova	2009	Bulgaria	105	E‐test	105	19.0	16.2	0	1.9	NA	5.8	NA	NA	NA	1.0	[72]
Agudo	2009	Spain	101	E‐test	101	49.2	32.8	0	0	NA	1.8	NA	NA	0	NA	[73]
Francavilla	2010	Italy	116	PCR	116	3.4	NA	NA	NA	NA	NA	NA	NA	NA	NA	[74]
Zevit	2010	Israel	174	E‐test	53	25.0	19.0	NA	NA	NA	NA	NA	NA	NA	NA	[75]
Kato	2010	Japan	61	E‐test	61	36.1	14.8	0	NA	NA	NA	NA	NA	NA	NA	[76]
Garcia	2010	Brazil	217	E‐test	45	27.0	13.0	4.0	0	NA	NA	NA	NA	NA	NA	[77]
Vecsei	2010	Austria	897	E‐test	153	34.0	22.9	0	0.9	NA	NA	NA	NA	0.9	NA	[78]
Vecsei	2010	Austria	143	E‐test	80	45.1	NA	NA	NA	NA	NA	NA	NA	NA	NA	[79]
Mansour	2010	Tunisia	48	E‐test	48	18.8	25.0	0	NA	NA	NA	NA	NA	NA	NA	[80]
Miendje	2011	Belgium	1527	DDT	1527	7.3	17.4	0	NA	NA	0.4	NA	NA	NA	0.3	[81]
Oleastro	2011	Portugal	1115	E‐test	1115	34.7	13.9	0	0	NA	4.6	NA	NA	NA	6.9	[82]
Kim	2011	Korea	33	E‐test	28	25.0	17.8	0	NA	NA	NA	NA	NA	NA	NA	[83]
Vecsei	2011	Austria	96	PCR	55	16.7	24.4	NA	NA	NA	NA	NA	NA	NA	NA	[84]
Scaletsky	2011	Brazil	217	E‐test	45	26.7	NA	NA	NA	NA	NA	NA	NA	NA	NA	[85]
Liu	2011	China	120	E‐test	73	84.9	61.6	0	0	13.7	NA	NA	NA	6.8	15.1	[86]
Nguyen	2012	Vietnam	240	E‐test	222	50.9	65.3	0.5	NA	NA	NA	NA	NA	NA	NA	[87]
Hojsak	2012	Croatia	3008	E‐test	382	11.9	10.1	0.6	NA	NA	NA	NA	NA	NA	NA	[88]
Milani	2012	Iran	395	DDT	112	9.5	81.1	23.8	4.8	NA	28.6	NA	NA	NA	NA	[89]
Megraud	2012	Europe	311	E‐test	311	31.8	25.7	0.3	0	NA	NA	NA	NA	NA	NA	[90]
Su	2013	China	17731	ADM	17731	21.5	95.4	0.1	NA	20.6	NA	NA	NA	NA	7.5	[91]
Ogata	2013	Austria	77	E‐test	77	19.5	40.0	10.4	0	NA	NA	NA	0	NA	NA	[92]
Seo	2013	Korea	58	ADM	33	18.2	27.3	24.2	15.2	NA	NA	NA	NA	NA	5.4	[93]
Goscinlak	2014	Poland	105	E‐test	105	33.3	44.8	NA	NA	NA	NA	NA	NA	NA	1.9	[94]
Montes	2014	Spain	143	E‐test	74	34.7	16.7	NA	NA	NA	NA	NA	NA	NA	NA	[95]
Gou	2014	China	73	E‐test	73	80.8	58.9	0	0	12.3	NA	NA	NA	6.8	1.4	[96]
Iwanczak	2014	Poland	9000	E‐test	222	20.2	27.4	0	0	NA	NA	NA	NA	NA	NA	[97]
Ogata	2014	Brazil	77	ADM	77	36.3	38.9	68.8	0	NA	NA	NA	0	NA	NA	[98]
Peretz	2014	Israel	41	E‐test	41	24.3	24.3	12.2	2.4	NA	NA	NA	NA	NA	NA	[99]
Karabiber	2014	Turkey	98	DDT	98	23.5	11.7	3.9	NA	NA	NA	NA	NA	NA	NA	[100]
Baars	2015	Netherlands	72	E‐test	72	7.2	10.4	NA	NA	NA	NA	NA	NA	NA	NA	[101]
Boyanova	2015	Bulgaria	40	E‐test	40	30.0	20.0	7.5	0.0	12.5	NA	NA	NA	4.3	0	[102]
Maleknejad	2015	Iran	169	DDT	21	13.8	8.26	12.3	10.1	NA	NA	NA	9.6	NA	NA	[103]
Manfredi	2015	Italy	66	E‐test	66	16.0	56.0	3.0	2.0	NA	NA	NA	NA	NA	NA	[104]
Macin	2015	Turkey	93	E‐test	93	30.1	48.4	0	0	NA	NA	NA	NA	NA	NA	[105]
Appak	2016	Turkey	200	PCR	200	9.5	NA	NA	NA	NA	NA	NA	NA	NA	NA	[106]
Regnath	2016	Germany	582	E‐test	582	23.2	28.7	0.8	NA	NA	NA	NA	NA	13.3	2.3	[107]
Correa	2016	Spain	136	PCR	111	47.7	NA	NA	NA	NA	NA	NA	NA	NA	NA	[108]
Lasso	2016	Colombia	133	PCR	133	8.0	NA	NA	NA	NA	NA	NA	NA	NA	NA	[109]
Schwarzer	2016	Sweden	209	E‐test	209	14.4	15.3	NA	NA	NA	NA	NA	NA	NA	NA	[110]
Butenko	2017	Slovenia	107	E‐test	107	23.4	20.2	1.0	0	2.8	NA	NA	NA	NA	2.9	[111]
Pastukh	2017	Israel	89	E‐test	89	38.0	8.0	12.0	8.0	2.0	NA	NA	NA	30	NA	[112]
Li	2017	China	5610	ADM	1746	16.38	75.20	0.06	NA	6.70	NA	NA	0.06	NA	2.17	[113]
Dargiene	2017	Lithuania	55	E‐test	55	21.8	25.0	0	NA	NA	0	NA	NA	8.3	NA	[114]
Kori	2017	Israel	95	E‐test	95	9.5	32.6	0	0	0	NA	NA	NA	NA	NA	[115]
Mahmoudi	2017	Iran	32	DDT	32	22.0	62.5	53.0	25.0	NA	37.5	NA	62.5	NA	NA	[116]
Serrano	2017	Chile	118	PCR	53	21.0	2.0	NA	NA	NA	NA	NA	NA	NA	NA	[117]
Luis	2018	Peru	285	PCR	49	79.6	NA	NA	NA	NA	NA	NA	NA	NA	NA	[118]
Silva	2018	Portugal	74	E‐test	74	23.3	3.3	0	NA	0	NA	NA	NA	NA	NA	[119]
Shu	2018	China	1390	ADM	545	20.6	68.8	0	NA	9.0	NA	NA	0	NA	2.9	[120]
Famouri	2018	Iran	102	E‐test	48	35.40	85.40	56.30	10.4	25.0	35.4	NA	NA	NA	NA	[121]
Mabe	2018	Japan	137	E‐test	21	29.0	NA	NA	NA	NA	NA	NA	NA	NA	NA	[122]
Jansson	2019	Sweden	1887	PCR	222	20.7	NA	NA	NA	NA	NA	NA	NA	NA	NA	[123]
Lu	2019	Taiwan	70	E‐test	70	22.9	21.4	2.9	0	8.3	NA	NA	NA	NA	NA	[124]
Guven	2019	Turkey	93	PCR	87	27.0	NA	NA	NA	15.0	NA	NA	NA	NA	NA	[125]
Moubri	2019	Algeria	112	E‐test	47	13.0	37.0	0	NA	NA	NA	NA	NA	NA	NA	[126]
Krzyzek	2020	Poland	126	E‐test	22	30.6	46.9	0	4.5	9.1	NA	NA	NA	NA	NA	[127]
Biernat	2020	Poland	108	E‐test	91	31.0	35.0	NA	NA	NA	NA	NA	NA	NA	NA	[128]
Zhang	2020	China	79	ADM	79	36.7	68.4	NA	NA	15.2	NA	NA	NA	NA	NA	[129]
Botija	2021	Spain	80	E‐test	80	44.9	16.3	2.0	0	7.9	NA	NA	NA	0	NA	[130]
Wang	2021	China	30	PCR	30	86.7	26.7	3.3	6.7	40.0	NA	NA	NA	NA	26.6	[131]
Miyata	2021	Japan	45	E‐test	45	71.1	NA	NA	NA	NA	NA	NA	NA	NA	NA	[132]
Li	2021	China	157	E‐test	87	55.2	71.3	0	0	18.4	NA	NA	0	60.9	NA	[133]
Thieu	2021	Vietnam	76	ADM	76	92.1	14.5	50.0	0	31.6	NA	NA	NA	NA	3.9	[134]
Helmbold	2022	Germany	124	E‐test	67	45.0	59.0	20.0	12.0	NA	31.0	NA	NA	22.0	16.0	[135]
Huang	2022	Singapore	70	DDT	70	30.0	27.5	7.1	NA	NA	NA	NA	NA	NA	2.9	[136]
Geng	2022	China	156	PCR	112	47.3	88.4	0	0	18.8	NA	NA	0	NA	10.7	[137]
Boyanova	2022	Bulgaria	106	E‐test	106	34.0	25.5	7.5	NA	NA	14.1	NA	NA	NA	6.6	[138]

### 2.3 Statistical Analysis

The pooled prevalence of Helicobacter pylori antibiotic resistance was calculated using the logit transformation of event rates, with corresponding 95% confidence intervals (CIs) to reflect precision. Effect sizes (proportions) and their CIs were subsequently back‐transformed to improve interpretability. Heterogeneity among studies was assessed using both Cochran's Q test and the I² statistic, with I² values of 25%, 50%, and 75% considered to represent low, moderate, and high heterogeneity, respectively, in line with Higgins et al. [139]. Due to anticipated methodological and population variability across included studies, a DerSimonian and Laird random‐effects model [140] was employed regardless of heterogeneity level, as recommended for meta‐analyzes of observational studies [141]. This model accounts for both within‐ and between‐study variance.

Subgroup analyzes were prespecified in the study protocol to examine differences in *H. pylori* antibiotic resistance across geographic regions and to evaluate temporal trends in pediatric resistance rates. All assumptions required for each statistical model were verified. Publication bias was assessed using funnel plot asymmetry, Egger's regression intercept test [142], and Begg's rank correlation test [143].

Statistical significance was defined as a two‐sided *p*‐value < 0.05, although interpretation emphasized effect sizes and CIs over *p*‐values, in accordance with contemporary reporting guidelines (e.g., Sterne et al. [144]). All analyzes were performed using Comprehensive Meta‐Analysis software version 2.2 (Biostat, Englewood, NJ, USA). Definitions of all statistical terms, abbreviations, and symbols are provided upon first mention to ensure clarity.

## 3 Results

### 3.1 Characteristics of Included Studies

**Figure 1:**
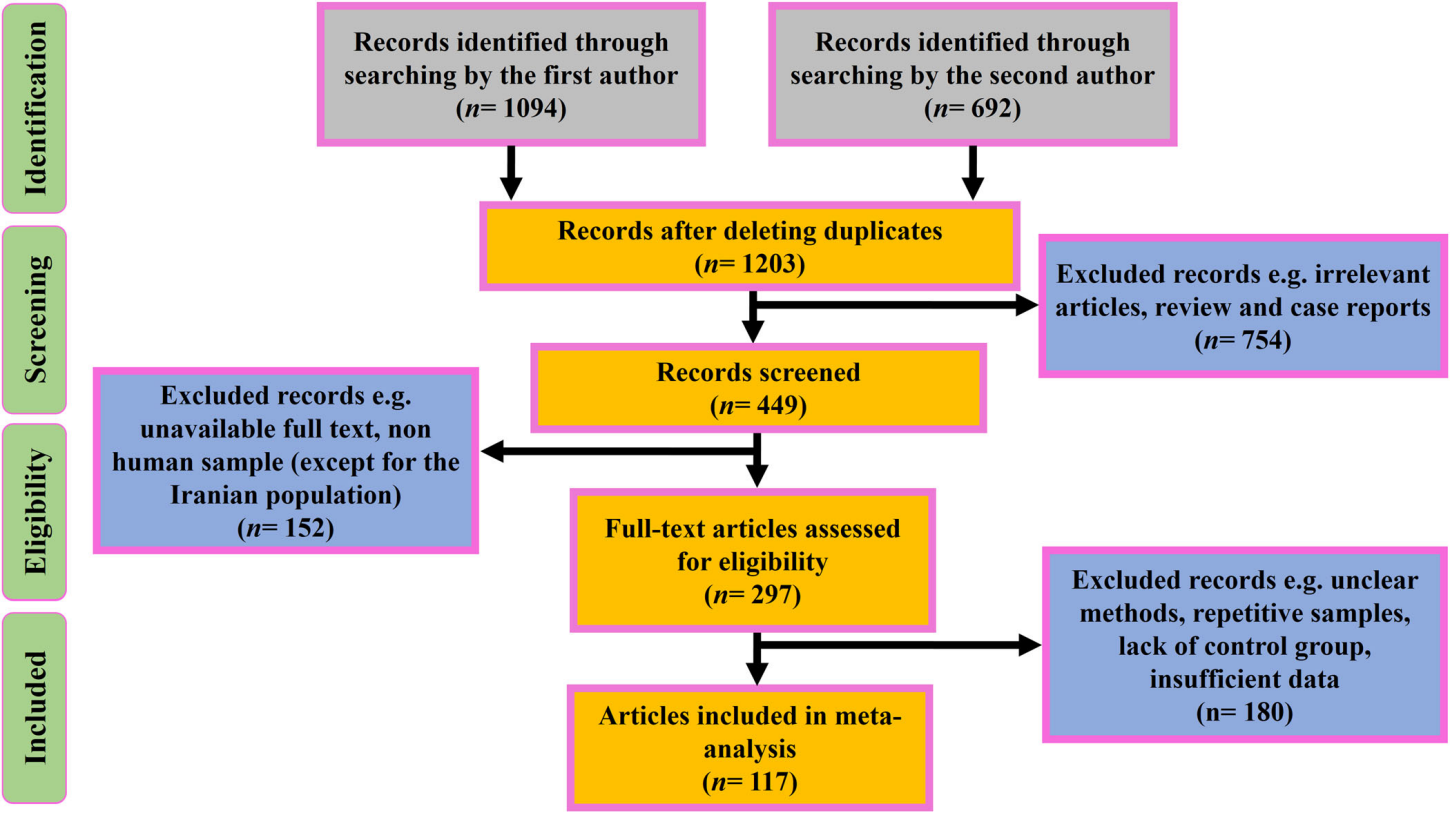
Flowchart of the literature search and study selection process for the systematic review. The diagram details the number of records identified through database searching, screening, eligibility assessment, and inclusion in the final analysis, following PRISMA guidelines.

Totally, 1786 records were identified throughout search in global databases. After evaluating title and abstract of all studies in the screening stage, 754 articles were excluded. After determining the compliance of the full‐text of relevant articles with our criteria, 117 articles were included in the current study (Figure 1). The most important reasons for omitting the articles were: (1) duplicate documents in databases; (2) review articles; (3) studies on adult population; (4) nonhuman investigations; (5) studies involving repetitive samples. Eligible studies had been conducted in Asia, Europe, North America, Latin America, Africa, and Oceania. A total of 57,143 patients were evaluated, of which 36,021 patients were *H. pylori*‐positive and 21,122 were *H. pylori*‐negative. Included studies were performed between 1992 and 2222. The *H. pylori* antibiotic resistance pattern had been determined using E‐test, agar dilution method (ADM), disk diffusion test (DDT), fluorescence in situ hybridization (FISH), and polymerase chain reaction (PCR).

### 3.2 Prevalence of *H. pylori* Primary Antibiotic Resistance

Our results suggested that the prevalence of *H. pylori* primary antibiotic resistance rates were 25.6% (95% CI: 22.7–28.8; *I* ^*2*^: 92.04; *p* value: 0.01; Begg's *p* value: 0.01; Egger's *p* value: 0.01) to clarithromycin, 30.9% (95% CI: 26.9–35.3; *I* ^*2*^: 93.76; *p* value: 0.01; Begg's *p* value: 0.01; Egger's *p* value: 0.01) to metronidazole, 2.5% (95% CI: 1.6–3.8; *I* ^*2*^: 91.97; *p* value: 0.01; Begg's *p* value: 0.59; Egger's *p* value: 0.01) to amoxicillin, 2.0% (95% CI: 1.3–3.0; *I* ^*2*^: 75.41; *p* value: 0.01; Begg's *p* value: 0.59; Egger's *p* value: 0.01) to tetracycline, 12.1% (95% CI: 8.2–17.6; *I* ^*2*^: 90.66; *p* value: 0.01; Begg's *p* value: 0.01; Egger's *p* value: 0.01) to levofloxacin, 6.9% (95% CI: 3.9–11.9; *I* ^*2*^: 90.17; *p* value: 0.01; Begg's *p* value: 0.03; Egger's *p* value: 0.01) to ciprofloxacin, 0.5% (95% CI: 0.00–0.07; *I* ^*2*^: 0.00; *p* value: 0.99) to nitrofurantoin, 1.9% (95% CI: 0.04–8.3; *I* ^*2*^: 93.24; *p* value: 0.01; Begg's *p* value: 0.39; Egger's *p* value: 0.03) to furazolidone, 9.1% (95% CI: 4.1–18.9; *I* ^*2*^: 93.38; *p* value: 0.01; Begg's *p* value: 0.06; Egger's *p* value: 0.01) to rifampin, as well as prevalence of MDR‐*H. pylori* was 4.5% (95% CI: 2.8–7.2; *I* ^*2*^: 80.77; *p* value: 0.01; Begg's *p* value: 0.01; Egger's *p* value: 0.01).

**Figure 2:**
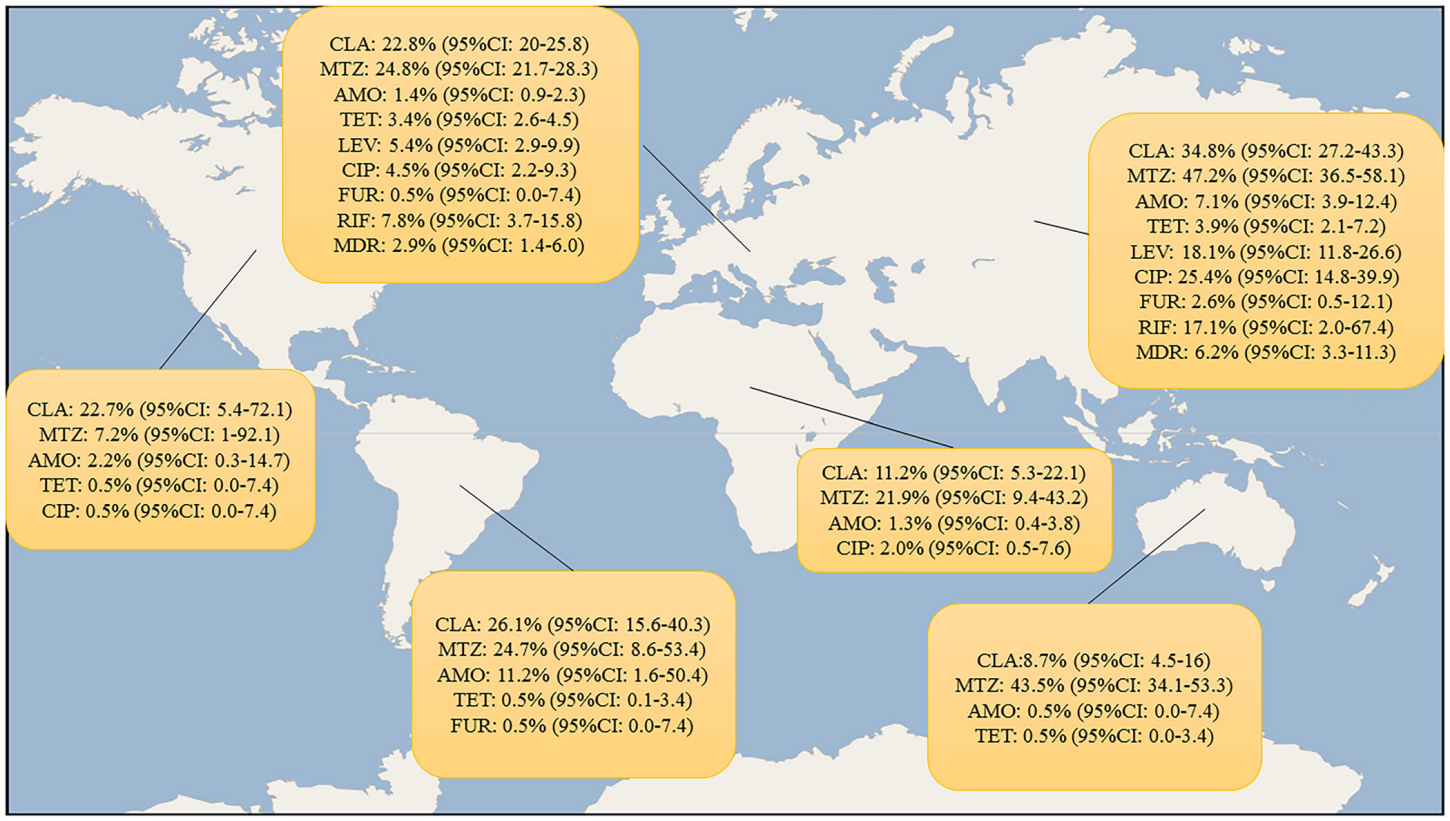
Geographical distribution of primary antibiotic resistance in *Helicobacter pylori* isolates from children. The map illustrates resistance rates to commonly tested antibiotics (e.g., clarithromycin, metronidazole, amoxicillin, and tetracycline) reported in included studies across different countries. Data represent the most recent available rates from each country.

Obviouslly, factors such as the pattern of antibiotic consumption in populations, genetic characteristics of circulating *H. pylori* strains in each region, and the previous history of antibiotic consumption are very different depending on the geographical area. Hence, through a subgroup analysis, we evaluated the prevalence of primary antibiotic resistance of *H. pylori* in children on different continents. According to the current analysis, the prevalence of primary MDR‐*H. pylori* is significantly higher in the Asian population than in Europeans (6.2% [95% CI: 3.3–11.3] vs. 2.9% [95% CI: 1.4–6.0], respectively). In addition, the prevalence of resistance to different classes of antibiotics is higher in Asian populations than on other continents; following Asia, antibiotic resistance is moderate in Latin America, Africa, and then Europe, and the lowest primary antibiotic resistance is in North America and Oceania (Figure 2).

### 3.3 Temporal Trends in *H. pylori* Primary Antibiotic Resistance in Children

To better understand how resistance to *H. pylori* treatment has evolved in pediatric populations, we analyzed primary antibiotic resistance rates across five time periods: before 2000, 2000–2005, 2006–2011, 2012–2017, and 2018–2022. Overall, the results indicate a concerning upward trend in resistance to multiple commonly used antibiotics (Table 3).

**Table 3:** Trend of *H. pylori* primary antibiotic resistance in children over the time.

Drug type	Before 2000		2000–2005		2006–2011		2012–2017		2018–2022	
	Rate	95% CI	Rate	95% CI	Rate	95% CI	Rate	95% CI	Rate	95% CI
CLA	24.9	10.5–48.4	22.1	17.5–27.5	23.3	17.9–29.6	23.0	18.6–28.2	39.5	30.5–49.2
MTZ	29.3	16.5–46.5	26.8	20.5–34.3	27.7	20.9–35.6	32.1	24.2–41.2	41.8	29.3–55.4
AMO	1.2	0.3–4.2	1.2	0.4–3.3	1.4	0.4–4.7	5.1	2.6–9.9	4.4	1.7–10.9
TET	0.5	0.1–2.0	1.7	0.9–3.3	2.2	1.4–3.6	2.8	1.4–5.7	3.8	1.8–7.8
LEV	0.5	0.0–7.4	25.1	1.1–90.9	13.7	8.2–21.9	7.4	3.9–13.7	15.7	10.8–22.2
CIP	0.6	0.1–2.6	4.5	2.6–7.7	5.2	3.4–7.9	24.4	11.2–45.0	26.1	15.5–40.4
FUR	NA	NA	NA	NA	1.8	0.2–16.6	4.6	0.5–32.7	0.5	0.1–2.4
RIF	NA	NA	NA	NA	2.0	0.3–11.4	10.8	5.0–21.7	20.0	4.2–58.6
MDR	NA	NA	1.2	0.2‐6.8	3.5	1.0–11.3	3.3	2.0–5.4	8.2	4.2–15.5

**Figure 3:**
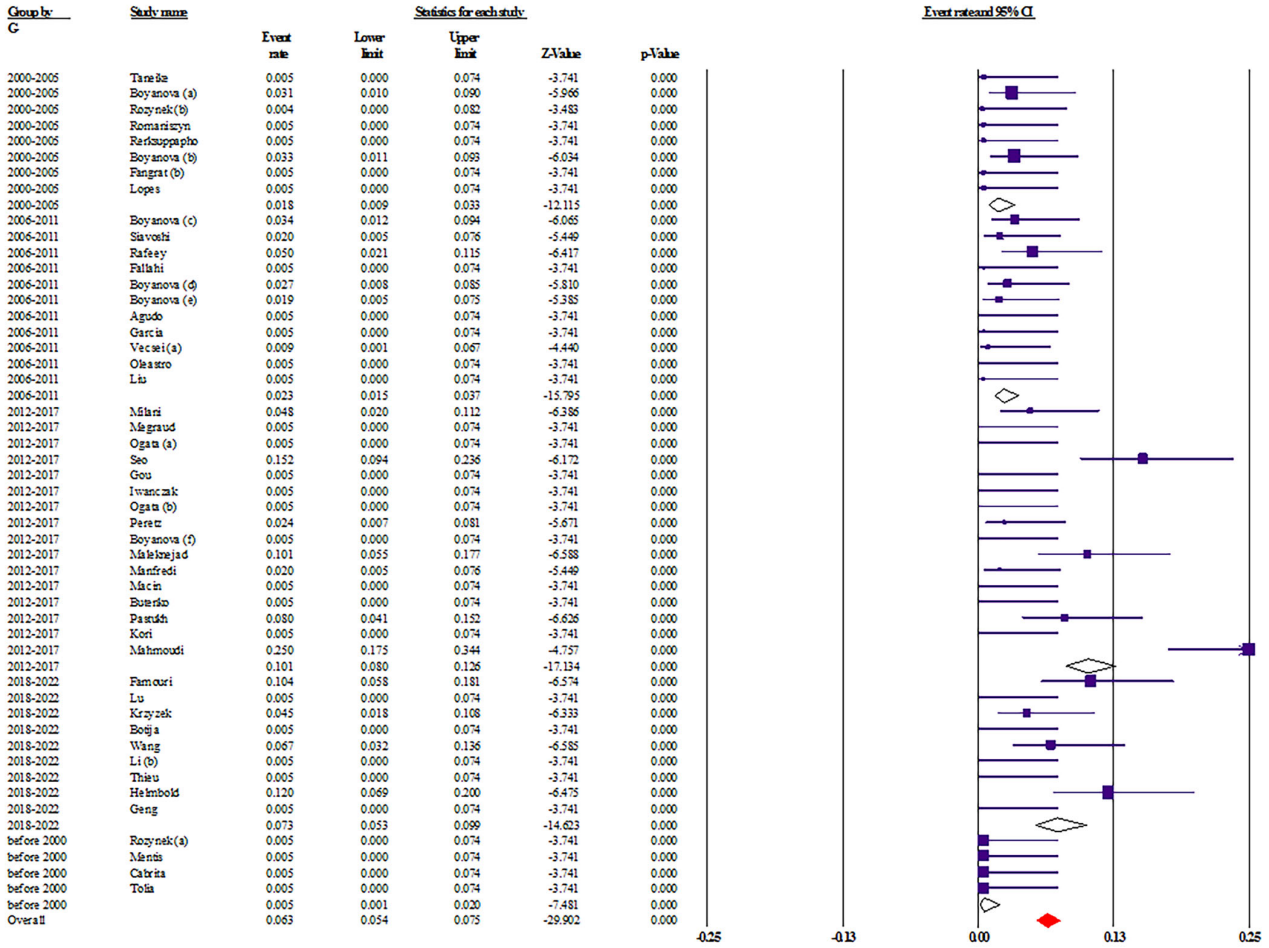
Temporal trend of primary tetracycline resistance in *H. pylori* infections among children from 2000 to 2022. The line graph shows the annual resistance rates (%) reported in eligible studies. Each data point corresponds to a study year; the trend reflects pooled estimates from multiple regions.

Clarithromycin (CLA), a cornerstone of first‐line therapy, showed relatively stable resistance rates (~22%–24%) between 2000 and 2017. However, resistance increased substantially to 39.5% (95% CI: 30.5–49.2) in the 2018–2022 period, suggesting a potential decline in clarithromycin‐based regimen efficacy in recent years. Metronidazole (MTZ) resistance remained consistently high, ranging from 26.8% to 41.8% over the study period. The latest estimate (2018–2022) indicates a resistance rate of 41.8% (95% CI: 29.3–55.4), underlining ongoing global challenges in metronidazole efficacy. Amoxicillin (AMO) resistance remained low initially but showed a notable rise from 1.4% (95% CI: 0.4–4.7) in 2006–2011 to 5.1% (95% CI: 2.6–9.9) in 2012–2017, with a slight decline to 4.4% (95% CI: 1.7–10.9) in the most recent interval. While still relatively low, the upward drift warrants attention. Tetracycline (TET) demonstrated low resistance across all periods, making it a viable option in rescue therapy. Tetracycline is one of the most effective antibiotics used in therapeutic regimens against *H. pylori* infection; this bacterium has a relatively low level of resistance to tetracycline, so it has attracted the attention of many gastroenterologists [145]. Nevertheless, a steady increase is evident from 0.5% (95% CI: 0.1–2.0) before 2000 to 3.8% (95% CI: 1.8–7.8) in 2018–2022, representing a 7.5‐fold increase, possibly linked to unregulated use (Figure 3). Fluoroquinolones, including levofloxacin (LEV) and ciprofloxacin (CIP), exhibited marked variability. LEV resistance peaked at 25.1% (95% CI: 1.1–90.9) in 2000–2005, likely due to sparse data, and stabilized around 15.7% (95% CI: 10.8–22.2) in the last time period. CIP resistance rose sharply from 0.6% (95% CI: 0.1–2.6) before 2000 to 26.1% (95% CI: 15.5–40.4) in 2018–2022. Other antibiotics such as furazolidone (FUR) and rifampin (RIF) were assessed in more recent years. FUR resistance fluctuated, while RIF resistance rose significantly from 2.0% (95% CI: 0.3–11.4) in 2006–2011 to 20.0% (95% CI: 4.2–58.6) in 2018–2022, though these results should be interpreted with caution due to wide confidence intervals and limited data. Importantly, multi‐drug resistance (MDR) also increased, from 1.2% (95% CI: 0.2–6.8) in 2000–2005 to 8.2% (95% CI: 4.2–15.5) in 2018–2022, emphasizing the growing complexity in managing pediatric *H. pylori* infections.

**Figure 4:**
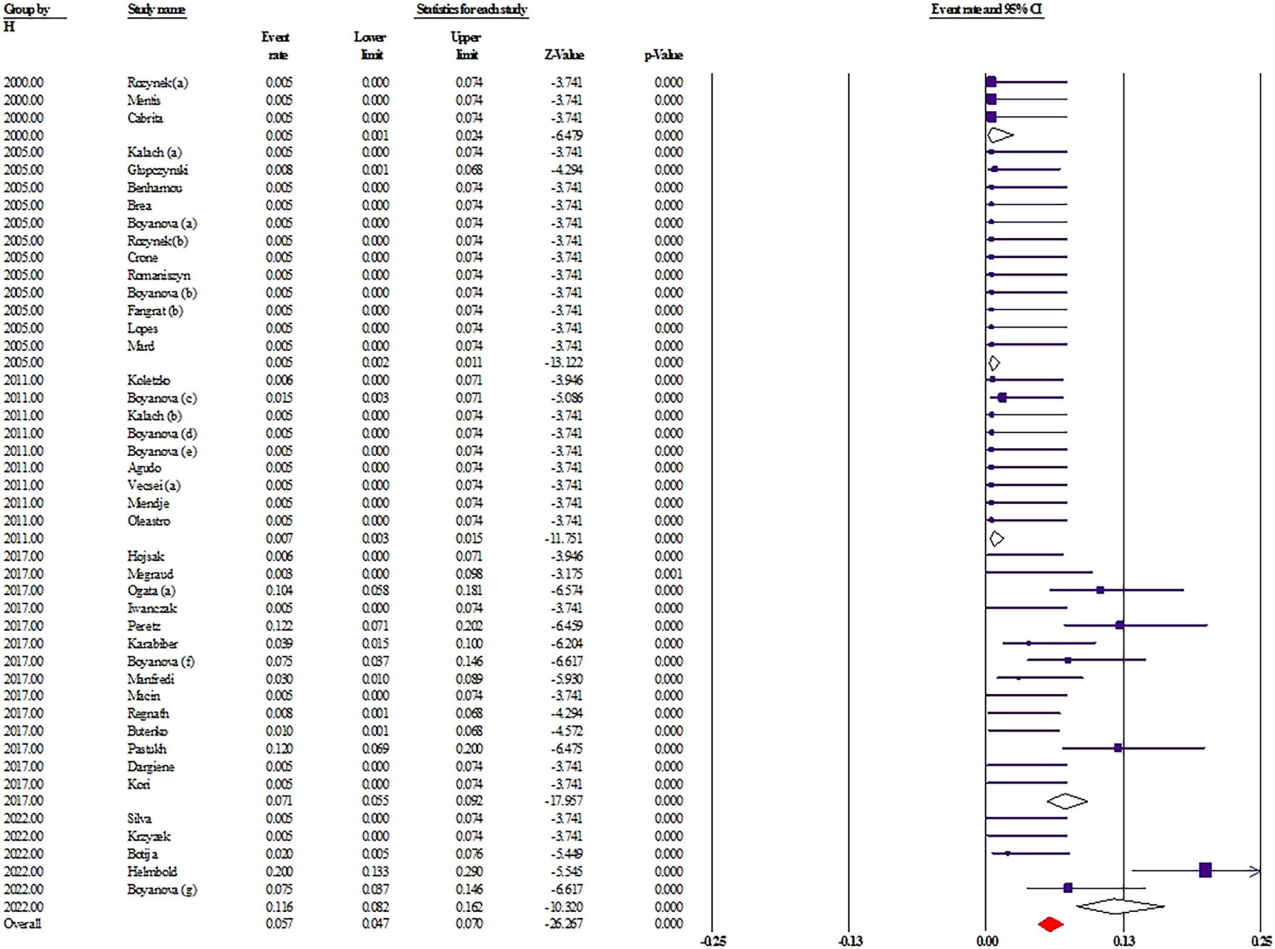
Temporal trend of primary amoxicillin resistance in *H. pylori* infections among children in European countries from 2000 to 2022. The graph displays resistance rates (%) over time based on reported data from eligible studies. Each point represents data from individual countries or study years, illustrating changes in prevalence across the continent.

Finally, we evaluated the changes in the initial antibiotic resistance of *H. pylori* in children over the past 22 years in different geographical areas (Table 4). Although discrepancies were observed in some years due to the lack of studies, we observed an increase in the primacy *H. pylori* antibiotic resistance rate in both hemispheres. For example, our analysis revealed that the primary resistance rate to amoxicillin in the European population has increased regularly over the years (Figure 4). Therefore, our study revealed that the primary antibiotic resistance rate of *H. pylori* strains in children is increasing in different geographical areas. Increased resistance burden, especially to clarithromycin, tetracycline and amoxicillin, is a serious threat and leads to increased treatment failure in children.

**Table 4:** Trend of *H. pylori* primary antibiotic resistance in various geographical regions over the past 22 years.

Drug type	Country	Before 2000		2000–2005		2006–2011		2012–2017		2018–2022	
		Rate	95% CI	Rate	95% CI	Rate	95% CI	Rate	95% CI	Rate	95% CI
CLA	Asia	NA	NA	35.1	22.6–50.1	23.9	10.6–45.4	26.3	13.6–44.9	49.2	34.7–63.8
	Europe	18.1	5.2–47.5	19.5	15.1–24.8	23.5	17.2–31.2	22.5	18.4–27.3	31.8	25.9–38.4
MTZ	Asia	NA	NA	19.2	6.5–44.9	47.3	29.0–66.4	61.0	41.0–77.8	53.3	32.8–76.2
	Europe	31.7	16.1–52.9	26.5	21.4–32.3	20.8	17.0–25.2	24.1	18.5–30.8	27.4	15.3–44.2
AMO	Asia	NA	NA	3.8	0.5–25.6	4.2	0.8–19.1	13.3	5.6–28.3	6.1	1.7–19.4
	Europe	0.5	0.1–2.4	0.5	0.2–1.1	0.7	0.3–1.5	3.4	1.9–6.2	3.9	1.1–13.1
TET	Asia	NA	NA	0.5	0.0–7.4	2.2	0.7–6.1	10.4	5.0–20.4	2.5	0.8–7.4
	Europe	0.5	0.1–2.4	1.9	0.9–4.0	2.1	1.1–3.8	1.3	0.5–3.3	5.2	1.5–16.4
MDR	Asia	NA	NA	1.2	0.2–6.8	15.1	9.3–23.5	4.3	2.2–8.3	7.0	2.5–18
	Europe	NA	NA	NA	NA	1.6	0.3–8.0	2.2	1.1–4.3	10.8	4.4–24.2

To combat and control the increase in antibiotic resistance in children, we need to review current treatment guidelines and try to introduce new therapeutic agents. Although the results of the present study were based on data analysis of 57,143 patients, we need more epidemiological studies with higher volumes around the world to accurately monitor changes in the pattern of *H. pylori* antibiotic resistance.

## 4 Discussion

The WHO designated *H. pylori* as a high‐priority antibiotic‐resistant bacterium in 2017, underscoring its significant threat to human health. Colonizing the stomachs of approximately 50% of the global population, this pathogen is implicated in over 95% of gastric cancer cases [146]. Consequently, widespread eradication of *H. pylori* infection in childhood holds the potential to mitigate the risk of severe gastrointestinal outcomes in later life [147]. However, the escalating challenge of antibiotic resistance renders the treatment of *H. pylori* infection in both adults and children increasingly difficult. A national survey in Japan reported a pediatric *H. pylori* triple therapy cure rate of approximately 71% [148], highlighting the existing challenges. Recent meta‐analyzes in children have further indicated that in regions with high resistance to clarithromycin and metronidazole, neither sequential nor triple therapy demonstrates clear superiority [149, 150, 151]. Updated guidelines from the Japanese Society for Pediatric Gastroenterology, Hepatology and Nutrition (JSPGHAN) caution against the effectiveness of a “test‐and‐treat” strategy for pediatric *H. pylori* eradication [152].

Based on the latest guidelines, administration of a PPI plus a high dose of two antimicrobial agents for 2 weeks can efficaciously eradicate *H. pylori* infection in children [15]. However, many differences in multiple drug‐resistant patterns in different parts of the world indicate that treatment protocols in Europe and North America may not be effective for other regions of the world [153]. However, several drawbacks such as lack of stewardship plan for *H. pylori*, excessive antibiotic usage, self‐medication, and administration of unnecessary antibiotics without the antimicrobial susceptibility testing (ATS), all intensify antibiotic resistance [11, 154]. Furthermore, problems like poor compliance, inadequate dose/duration, CYP2C19 polymorphism, ineffective penetration of antibiotic, and antibiotic destruction in acidic gastric environment also cause treatment failure [119, 126, 155]. Launching a robust network of national and global surveillance systems for tracking antibiotic prescribing and continuous monitoring of changes in antibiotic resistance patterns in different parts of the world can be useful for purposes such as designing more effective therapeutic guidelines and controlling the emergence of antibiotic resistance [156]. As far as we know, no comprehensive study has been conducted to evaluate the antibiotic resistance of *H. pylori* in children.

This study was the first systematic review and meta‐analysis that evaluated the resistance rate and trend of primary antibiotic resistance in children in the last 22 years; our results suggested that the rate of antibiotic resistance is worrying. The highest resistance rate was in the Asian population, while the lowest antibiotic resistance rate was found in North America and Australia. We also showed that the antibiotic resistance rate has steadily increased over the last 22 years, particularly in Asia and Europe. Our analysis revealed a gradual increase in clarithromycin resistance from 24.9% before 2000 to 39.5% in 2022. In the subgroup analysis, we found that clarithromycin resistance has been increased in both Asian countries and European pediatric population in recent years (35.1%–49.2% and 18.1%–31.8%). This significant increase might be due to the increase in macrolides consumption. In accordance with our results, in a recent study conducted by Megraud et al. on 1211 European adult patients, they found that the resistance rates to clarithromycin, levofloxacin, and metronidazole were 21.4%, 15.8%, and 38.9%, respectively [157]. In a review article by Thung et al., the global resistance to clarithromycin in countries such as Japan, Italy, China, Turkey, Sweden, and Taiwan was reported 30%, 30%, 50%, 40%, 15%, and 15%, respectively [12].

Metronidazole resistance rate has increased about twofold in the last 22 years. It seems that Asian countries play a significant role in increasing the global resistance of *H. pylori* to metronidazole. Subgroup analysis found that the trend of resistance to metronidazole in Asian children has increased from 19.2% between 2000 and 2005 to 53.3% in 2022, while this trend has experienced even a slight reduction among European children (31.7%–27.4%). Excessive use of metronidazole for parasite infection, pelvic inflammatory disease (PID) as well as dental infection in Asian developing countries has significantly increased antibiotic resistance rate in these geographical areas [158, 159]. In a recent meta‐analysis conducted by Kuo et al., the rate of resistance to metronidazole among *H. pylori* strains isolated from Asian adults was reported about 44%; they showed that metronidazole resistance in Asian low‐income countries is much higher than that in countries with highest socioeconomic status, such as Japan [160]. However, with the increase in resistance to metronidazole in Asian countries, it seems that the administration of this antibiotic for *H. pylori* infection is not reasonable and should be stopped.

We observed a remarkable increase in resistance to amoxicillin, tetracycline and levofloxacin between 2000 and 2022 (1.2%–4.4%, 0.5%–3.8%, as well as 0.5%–15.8%, respectively). According to subgroup analysis, trend of resistant to these classes of antibiotics steadily increased in both Asian and European population over the past 22 years ago. In addition, ciprofloxacin resistance also significantly increased from 0.6% in 2000 to 26.1% in 2022. According to recent studies on the population of Taiwan and Europeans, the use of fluoroquinolones is significantly associated with increased resistance of *H. pylori* isolates to these group of antibiotics [90, 161].

According to Van Boeckel et al. study, global consumption of fluoroquinolones and macrolides has increased by 64% and 19% in recent years, respectively, which in turn has increased the resistant burden to these classes of antibiotics throughout of the world [162]. Although the resistance to amoxicillin and tetracycline in our study was not very high, the increasing trend of resistance to these antimicrobial agents in the world is considered very worrying, especially in Asian and European countries. Easy access to these antibiotics and wide use for treatment of various bacterial infections justifies a trend towards increasing resistance in recent years [163, 164]. Our results indicated that resistance to rifampin, furazolidone, and nitrofurantoin was relatively low. Evaluation of trend also showed contradictory results. Low resistance to these antibiotics may be due to the fact that they are usually not recommended due to their side effects such as toxicity and carcinogenic properties [92]. However, recent studies have shown the efficacy of these antibiotics in increasing *H. pylori* cure rate, so that they may replace tetracycline [165].

Although treatment outcomes differ between children and adults, Savoldi et al. in their recent study showed that the rate of primary and secondary levels of *H. pylori* antibiotic resistance to clarithromycin, metronidazole and levofloxacin in adults was higher than 15%, which is similar to our results; they also showed that the trend of antibiotic resistance has been increased among adults in recent years [151]. In a recent meta‐analysis, Khurana et al. introduced the most effective treatment regimens for *H. pylori* infection in children in developed countries as follows: nitroimidazole and amoxicillin, 2–6 weeks; clarithromycin, amoxicillin and a PPI, 1–2 weeks; a macrolide, a nitroimidazole and a PPI or bismuth, amoxicillin and metronidazole, 2 weeks [166]. Although recent studies confirm the efficacy of standard first‐line triple therapy in areas with less than 15% resistance, a steady increase in resistance in recent years will increase the risk of treatment failure in children. Bismuth based quadruple therapy should be considered as an alternative first‐line treatment choice for areas with high levels of antibiotic resistance. Graham et al. showed that bismuth‐containing regimens can increase the eradication rate of *H. pylori* infection by 30%–40% [167]. Our study had several limitations: (1) the protocol for this systematic review and meta‐analysis was not pre‐registered; (2) there is remarkable heterogeneity between included studies (study periods, various geographical regions, ethnicity, gender and age distribution, variety of genetic characteristics of *H. pylori* strains, history of antibiotic consumption, and the methods that evaluated antibiotic resistance can be effective as a source of heterogeneity); (3) the presence of a significant publication bias; (4) information on antibiotic resistance was not available in many countries, especially poor developing countries. Thus, current findings should be interpreted with more caution, and we require further larger investigation with appropriate study design to confirm the validity of the present findings.

## 5 Conclusions

The present study was the first comprehensive review and meta‐analysis on primary *H. pylori* antibiotic resistance in children. Our results showed that the primary antibiotic resistance rate to clarithromycin, metronidazole, levofloxacin and ciprofloxacin was high. The frequency of primary resistance of MDR‐*H. pylori* was calculated at about 4.5%. We showed that the trend in *H. pylori* antibiotic resistance has increased in most regions in the last 22 years. The rate of increase in antibiotic resistance in Asian countries is higher in regions with low socioeconomic status than in other regions. Increased antibiotic resistance of *H. pylori* in children is very worrying, because not eradicating this bacterium in childhood can be associated with peptic ulceration or gastric cancer in adulthood. The pattern of antibiotic resistance largely depends on national antibiotic consumption. In addition, administration of abundant antibiotics can also increase the emergence of MDR strains. Thus, performing mechanisms such as AST before administrating of treatment regimen, tailored therapy, forming a global network for monitoring changes in antibiotic resistance in different geographical areas, all lead to effective treatment of *H. pylori* and control of the annual increase in antibiotic resistance in children.

## Author Contributions

**Shaho Menbari:** writing – original draft, conceptualization. **Sara Kamal Shahsavar:** writing – original draft, conceptualization. **Masoud Keikha:** writing – original draft, conceptualization, methodology, writing – review and editing. **Mohsen Karbalaei:** writing – original draft, writing – review and editing, conceptualization, methodology.

## Ethics Statement

This study is based entirely on published data and did not involve any individual patient data; therefore, ethical approval and informed consent were not required.

## Conflicts of Interest

The authors declare no conflicts of interest.

## Transparency Statement

The lead author Masoud Keikha, Mohsen Karbalaei affirms that this article is an honest, accurate, and transparent account of the study being reported; that no important aspects of the study have been omitted; and that any discrepancies from the study as planned (and, if relevant, registered) have been explained.

## Data Availability

The data that support the findings of this study are available from the corresponding author upon reasonable request. All authors have read and approved the final version of the article (Mohsen Karbalaei) had full access to all of the data in this study and takes complete responsibility for the integrity of the data and the accuracy of the data analysis.
